# Kilohertz droplet-on-demand serial femtosecond crystallography at the European XFEL station FXE

**DOI:** 10.1063/4.0000248

**Published:** 2024-04-17

**Authors:** Samuel Perrett, Alisia Fadini, Christopher D. M. Hutchison, Sayantan Bhattacharya, Cade Morrison, Oleksii Turkot, Mads Bregenholt Jakobsen, Michael Größler, José Licón-Saláiz, Florian Griese, Samuel Flewett, Joana Valerio, Joachim Schulz, Mykola Biednov, Yifeng Jiang, Huijong Han, Hazem Yousef, Dmitry Khakhulin, Christopher Milne, Anton Barty, Jasper J. van Thor

**Affiliations:** 1 Department of Life Sciences, Faculty of Natural Sciences, Imperial College London, London SW7 2AZ, United Kingdom; 2 Central Laser Facility, Rutherford Appleton Laboratory, Didcot OX11 0QX, United Kingdom; 3 European XFEL, Holzkoppel 4, 22869 Schenefeld, Germany; 4 Center for Data and Computing in Natural Sciences (CDCS), Notkestrasse 10, D-22607 Hamburg, Germany; 5 Section for Biomedical Imaging, University Medical Center Hamburg-Eppendorf, D-20246 Hamburg, Germany; 6 Institute for Biomedical Imaging, Hamburg University of Technology, D-21073 Hamburg, Germany

## Abstract

X-ray Free Electron Lasers (XFELs) allow the collection of high-quality serial femtosecond crystallography data. The next generation of megahertz superconducting FELs promises to drastically reduce data collection times, enabling the capture of more structures with higher signal-to-noise ratios and facilitating more complex experiments. Currently, gas dynamic virtual nozzles (GDVNs) stand as the sole delivery method capable of best utilizing the repetition rate of megahertz sources for crystallography. However, their substantial sample consumption renders their use impractical for many protein targets in serial crystallography experiments. Here, we present a novel application of a droplet-on-demand injection method, which allowed operation at 47 kHz at the European XFEL (EuXFEL) by tailoring a multi-droplet injection scheme for each macro-pulse. We demonstrate a collection rate of 150 000 indexed patterns per hour. We show that the performance and effective data collection rate are comparable to GDVN, with a sample consumption reduction of two orders of magnitude. We present lysozyme crystallographic data using the Large Pixel Detector at the femtosecond x-ray experiment endstation. Significant improvement of the crystallographic statistics was made by correcting for a systematic drift of the photon energy in the EuXFEL macro-pulse train, which was characterized from indexing the individual frames in the pulse train. This is the highest resolution protein structure collected and reported at the EuXFEL at 1.38 Å resolution.

## INTRODUCTION

X-ray Free Electron Lasers (XFELs) have proven to be a powerful tool for studying chemical and biological mechanisms through crystallography.^1–7^ XFELs deliver femtosecond pulses with peak brilliance over a billion times more intense than that of their synchrotron counterparts.^8^ The short duration of the pulses allows tracking of structural and coherent dynamics on a sub-picosecond level,^1,9^ for many photoreactions where the initial product-determining reaction step occurs. However, due to the high peak brilliance, these X-rays destroy the sample upon exposure. The consequent necessity to replace the crystal after each shot means that XFEL crystallography is typically conducted using a serial approach, where crystals are rapidly introduced and replaced for each x-ray pulse.^10^ High-resolution structure determination through serial femtosecond crystallography (SFX) requires large datasets ∼10 000–100 000 frames. This translates to significant sample consumption, which can further increase by up to two orders of magnitude for sample delivery techniques with low hit rate, a factor that is prohibitive for targets that cannot be easily produced in such quantities.

The advent of superconducting XFELs, such as the European XFEL (EuXFEL) and Linac Coherent Light Source (LCLS) II-HE, which operates at megahertz (MHz) repetition rate, promises faster data collection and better signal-to-noise for serial crystallography.^11^ EuXFEL is currently the highest repetition rate XFEL, capable of delivering up to 27 000 pulses per second in a “burst mode,” with a train duration of 600 *μ*s at a repetition rate of 10 Hz.^12^ LCLS-II is expected to start accepting users in the near future with a true megahertz repetition rate. Once operational, it is anticipated that the x-ray source will not be the limiting factor in SFX measurements. Instead, the focus will shift to improving detector technologies, optical excitation methods, and sample delivery systems to fully leverage the capabilities of the latest generation of x-ray sources.^13,14^

Crystal delivery methods for SFX experiments can broadly be split into two categories. First, fixed-target methods place crystals on solid supports and move them through the beam. These boast high hit rates but possess their own limitations, such as device loading time and potential sample evaporation issues.^15–19^ Although fixed-targets can achieve high hit rates, they cannot keep up with MHz repetition rate of the latest FELs due to physical limits on translating a fixed target and the finite physical size. Second, liquid phase injectors, such as grease injectors, offer low sample consumption and high hit rates.^20,21^ However, grease injectors are not suitable for MHz XFELs due to their slower delivery speeds and inability to refresh the sample volume. An alternative liquid target is the gas dynamic virtual nozzle (GDVN), which is the only method at present capable of delivering samples at >100 Hz repetition rates.^22,23^ GDVNs deliver a high velocity stream of crystal slurry to the XFEL beam focused by helium gas. Variations like double flow-focusing nozzles (DFFNs)^24^ and co-flow systems offer improved stability and reduced waste. The low hit rate and rapid jet velocities required to replace the crystals on a shot-to-shot basis, on the other hand, lead to an extremely low index rate of all frames (<5%). These issues are exacerbated at the EuXFEL, where, with continuous GDVN injection, the majority of the sample arrives between the trains and is consequently wasted. The very high sample consumption renders MHz SFX unfeasible for many biologically relevant targets that are not available in the quantities required, typically grams. Sample delivery at FELs has been previously presented and discussed in detail.^25–28^ Droplet-on-demand (DoD) injection methods have proved very successful at delivering high data rates with small sample consumption at ∼100 Hz FELs.^1,29–34^ While the repetition rate of this method is physically capped by the acoustic wave generation and dampening speed of the injector, this technology can be theoretically be advanced to MHz operation.^35,36^ The feasibility of this method at the latest high repetition rate FELs is yet to be demonstrated past 100 Hz.

Here, we report kHz droplet-on-demand serial crystallography, yielding rates of indexed crystals per second comparable with published MHz GDVN experiments,^27,37–40^ with a two order of magnitude reduction in sample consumption. The data were collected at the Femtosecond X-ray Experiments (FXE) beamline of the EuXFEL, marking the first report of serial protein crystallographic measurements at this beamline. The use of the Large Pixel Detector (LPD) in protein crystallography for the first time resulted in achieving higher resolution limits than any previous protein crystallographic experiments at the EuXFEL. The implementation of supervised learning algorithms for masking, along with efficient data handling techniques, streamlined the process of managing protein SFX data with this detector. By developing and optimizing these processing methods, we were able to maximize data quality from the LPD, leading to the highest resolution protein structures obtained from the EuXFEL. Our work demonstrates the feasibility of collecting high-resolution protein crystallography data at MHz FELs with sample volumes not uncommon in biochemistry laboratories, lowering the barrier to entry for the emerging field of SFX.

## METHODS

### Droplet-on-demand injection design

Droplet-on-demand (DoD) injection techniques utilize a piezo actuator to create an acoustic wave in the sample medium. This negative pressure wave travels down the nozzle, pushing the sample's meniscus out of the orifice. The deformation of the meniscus then breaks off, forming a droplet due to surface tension forces. The actuation of the piezoelectric material, controlled by the supplied electrical waveform, allows for tuning of the acoustic wave and the resultant droplet characteristics. Additionally, the properties of the sample medium, such as crystal density and viscosity, are crucial factors influencing droplet formation.^41,42^ Solutions with viscosities between 0.5 and 40 cP can be jetted at these higher rates. We have jetted solutions of PEG 6000 (25%) and sodium malonate (3.2M), common carriers for biological crystals. The DoD injector was an adapted inkjet printing system manufactured by MicroFab Technologies, consisting of the JetDrive controller (CT-M3-02), pressure controller (CT-PT-21-1), printhead assembly (PH-47-AB), and 80 *μ*m microdispensing device (MJ-AB-15-80 DLC). A schematic diagram of the setup can be found in supplementary information Fig. A.

The major practical concern of EuXFEL is the macro-pulse (train) structure of the X-rays.^43^ The precise timing control of the DoD method significantly reduces sample usage by allowing the injection to be paused during the >99% of total time that the X-ray is idle. However, for a usable data rate in SFX experiments (exceeding 10 Hz), it is crucial to achieve diffraction from multiple pulses within a single train. To meet this requirement, droplets must be generated at frequencies ranging from kHz to MHz and contain sufficient crystal density.

**FIG. 1.**
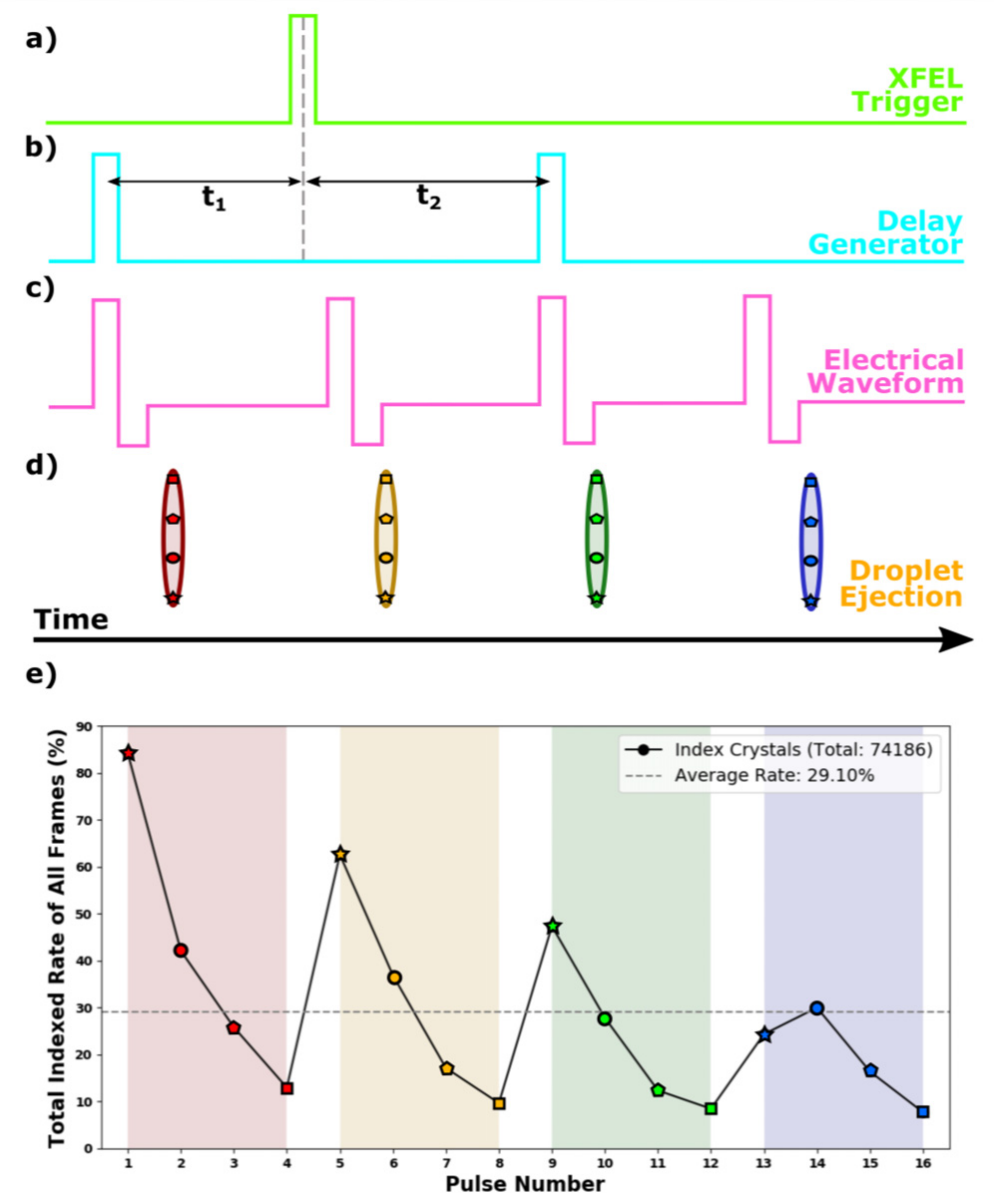
Knackwurst schematic depicting the process of triggering and generating waveforms for droplet formation. (a) The sequence begins with a trigger from the Free-Electron Laser (FEL). (b) This trigger is followed by two additional triggers generated by a delay generator. The first of these delayed triggers occurs before the original FEL trigger. (c) These two delayed triggers are then fed into an electrical waveform generator, which is electronically controlled by MicroFab software. The electrical waveform generator produces a specific voltage pattern: initially, a large positive voltage of +80 V is applied, followed by a short negative voltage of −40 V. This voltage pattern is key in forming a droplet with a Knackwurst shape. Each Transistor-Transistor Logic (TTL) signal from the delay generator is responsible for creating two electrical waveforms, resulting in a total of four waveforms. These four electrical waveforms are all independently controlled and are crucial in the operation of the piezo-actuator. (d) The piezo-actuator, in response to these waveforms, ejects four droplets. Each of these droplets is then hit four times by the XFEL pulses. (e) The resulting hit rate for each hit within the Knackwursts. A decline in the total indexing rate is evident as the hit rate decreases, attributed to increased instability with each additional hit. A slight reduction from Knackwurst-to-Knackwurst is observed due to incomplete dampening of the previous acoustic wave affecting subsequent Knackwurst ejection. The average hit rate over the collection period was 29%. All FEL pulses hit a Knackwurst none missed between the injection.

The primary challenge in this process arises from fluid dynamics. Specifically, the fundamental limitation to the injection rate stems from the damping effect of the acoustic wave in the sample medium and the speed of droplet ejection.^36^ Ensuring the dampening of the previous acoustic wave is critical for stable jetting, as it guarantees that each subsequent droplet is unaffected by its predecessor. To maximize the intra-pulse injection rate, our method uses a combination of two distinct approaches. First, high voltage long electrical waveforms allow for an ejection of an elongated droplet, here named “Knackwurst” (a Hamburg sausage). The Knackwurst can then be hit multiple times over the train by matching the pulse rate to the droplet velocity. The longer, elongated shape of these droplets affords a larger cross section than a smaller spherical droplet. Variations in the crystal density, precipitation, and debris resulting from droplet explosion over the data collection period can induce positional changes in a droplet within the pulse sequence. The increased volume of the Knackwurst droplets provides a larger margin of error, contributing to a sustained high hit rate. Second, four of these Knackwurst droplets are ejected over a train, each triggered by a distinct electrical waveform (Fig. 1). The XFEL had an intra pulse repetition rate of 47 kHz, meaning that data were collected at an effective repetition rate of 160 Hz. We note that future work could extend this repetition rate, as the MicroFab injector specifications allow for producing droplets at 45 kHz, which could be subjected to multiple hits.

The four Knackwursts were generated as follows. A Transistor-Transistor Logic (TTL) trigger from the XFEL was routed through a delay generator, which produced two independent TTL triggers. The first trigger was timed to precede the XFEL TTL, initiating the first Knackwurst injection before the first XFEL pulse—effectively syncing it with the preceding FEL pulse. The minor shot-to-shot drift is negligible for these injection timescales. These two independent triggers from the delay generator were used to trigger the JetDrive III signal generator. For each trigger, two separate voltage signals were sent to the piezo-actuator printer assembly (Fig. 1). Consequently, the printer assembly received four electrical waveforms, resulting in the output of four consecutive Knackwursts. A rapid-rise-and-fall waveform, which quickly ascends (5 *μ*s) to a high positive voltage (70–90 V), briefly (5 *μ*s) maintains this level and then swiftly descends (5–10 *μ*s) to a negative value (−40 V) for a short period (10–20 *μ*s), before rapidly returning (5 *μ*s) to 0 V. This waveform generates the elongated droplets (knackwursts) at a high repetition rate.

**FIG. 2.**
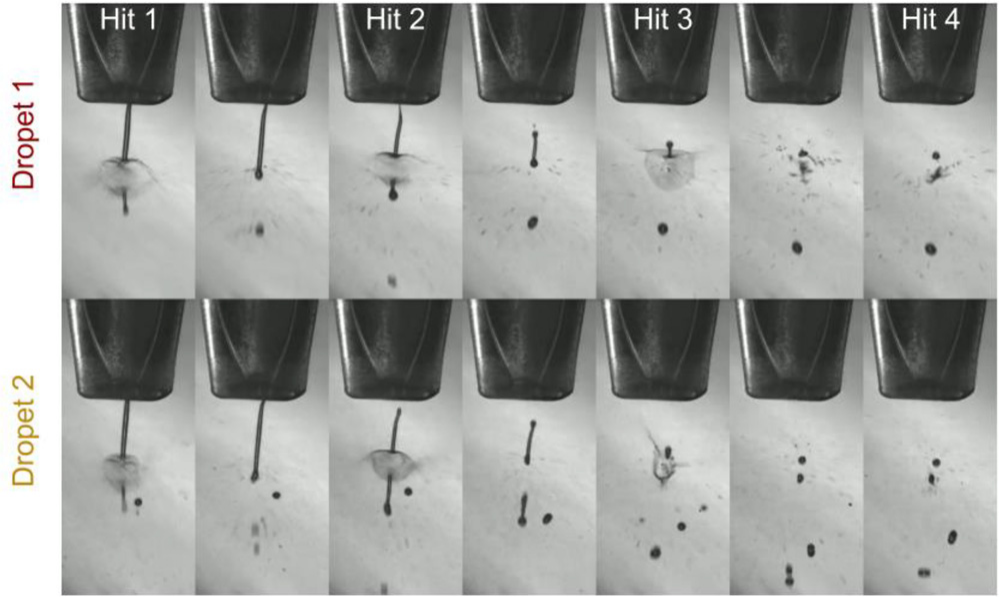
Droplet-on-demand injection scheme. Top: Images from the first Knackwurst (elongated droplet) ejection sequence, wherein the Knackwurst undergoes four successive hits (labelled hit 1 to 4), each marked by a notable explosion. Each frame is shown at intervals of 10.5 *μ*s. Bottom: A similar sequence for the second Knackwurst, in total four Knackwursts were ejected, each experiencing four hits. Consequently, 16 X-ray pulses were delivered over the macro-pulse at a frequency of 47 kHz. Note images at higher flux for droplet alignment and only display first two out of four Knackwursts.

A crystallization buffer was used to partially fill the sample reservoir and all tubing. Subsequently, the crystalline sample (crystal density 18%) was reverse-loaded through the nozzle by applying a −0.5 psi backing pressure, serving as a final filtration step. Typical 100 *μ*l of sample was loaded, under stable conditions enough for ∼2 h. Care was taken to avoid introducing any air bubbles into the system. Anecdotally, it was found that maintaining a small air bubble just inside the 80 *μ*m exit hole of the jet did aid in jetting less viscous buffers. This is assumed to be due to a dampening effect that disrupts the acoustic wave traveling in reverse direction after droplet ejection. A slight backing pressure was applied throughout jetting at −0.2 psi, to maintain the meniscus flush without the orifice of the nozzle. Jetting was maintained during the hutch closing procedure to prevent clogging. However, to minimize sample wastage, a separate waveform scheme operating at 10 Hz was employed. The sample concentration was adjusted to approximately 3–6 × 10^7^ Crystals/ml, varying with the sample, to ensure consistent hits without clogging the nozzle. Live feedback on the hit rate was provided by EXtra-Foam, with a consistent average hit rate of 30%–50% being achievable after some optimization (supplementary information D).^44^ The sample was replenished when the hit rate dropped below 15% and continued operation without disruption was typically possible for 1-2 h. Fine tuning of trigger delays and spatial position of the jet was done via the live feedback, and this was adjusted over the run accounting for any debris buildup on the jet. Live imaging of the jetting process, as shown in Fig. 2, was conducted using a FLIR Blackfly S (BFS-U3-04S2M-CS) camera focused on the jet. Additionally, a small LED on a variable delay was employed as a strobe to record the droplet injection at various time delays (Fig. 2). Initially, the alignment of the DoD was achieved by increasing the X-ray flux to visually observe the explosion. Subsequently, data collection was conducted at a lower flux to minimize any shockwave-induced crystal deformities, which have been reported at high repetition rates.^45^

The four distinct Knackwursts allow examination of potential shock-wave-induced alterations in crystal dimension and quality of diffraction. GDVN sample delivery at 1.1 MHz repetition rates have shown no such differences.^37^ However, at the higher repetition rate of 4.5 MHz, there has been significant shock induced damage reported.^45^ The GDVN and DoD injection conditions differ in the thickness of the jet as well as the velocity. A distance of ∼100 *μ*m separates subsequent hits within a Knackwurst (total length ∼600 *μ*m), which corresponds to a volume of 0.09 nl. We note that this is difficult to estimate with varying dynamics of the droplet formation and injection due to surface tension and perturbation by the X-rays. With the DoD delivery method operating at 47 kHz, we observed no shot-to-shot changes within a single droplet, as discussed from the crystallographic results below. Figure 2 displays the images of the injection scheme with successive hits on the same droplet demonstrating a decreasing hit rate for shots within each Knackwurst, attributed to the random instability caused by the explosion from the previous hit(s). However, when averaged over the entire injection scheme, each pulse demonstrated an average indexing rate of 29% (of all frames), which is significantly higher compared to other sample delivery methods [GDVN ∼ 5% (Ref. 46)]. This substantial increase in the hit rate, in comparison with GDVN, enables a higher effective data rate, or number of indexed patterns per second, surpassing all but one of the previous lysozyme MHz SFX experiments conducted at the EuXFEL, shown in Table II.^37–40,47^

### FXE data collection instrumentation

Data were collected at Femtosecond X-ray Experiments (FXE) endstation at the European XFEL.^48,49^ Sample delivery was as described as above and in the main text. The sample environment enclosure was purged with helium before data collection, beyond this there was approximately ∼15 cm of air before the detector. The photon energy of the XFEL was ∼9.3 keV, and up to 2.6 mJ pulse energy, 16 pulses were provided within the train at a repetition rate of 47 kHz at 20% transmission. The x-ray beam spot size was 10 × 11 *μ*m at interaction region (supplementary Fig. P). The data collection was performed using the Large Pixel Detector (LPD),^50^ which features a pixel size of 500 × 500 *μ*m and three gain stages, at a detector distance of 0.235 m. A number of additional data processing steps were required nonstandard to other detectors, which are discussed below.

### Data analysis

EXtra-Xwiz^51^ was used to process data with CrystFEL 10.2.^52–54^ Peak finding was performed by “peakfinder8”^55^ and integration carried out by XGANDALF (extended gradient descent algorithm for lattice finding),^56^ with multi crystal indexing turned on. Additionally, the nonstandard “integration = rings-cen” was used to recenter the indexing area, with ring integration radii of 1,2,3 due to the large pixels and detector size of the LPD. Several iterations of “geoptimiser” and “detector-shift” were employed to refine the detector geometry. Due to the LPD's large size and the large sample to detector distance, errors in detector geometry have a more pronounced effect on indexing when compared to other crystallographic detectors at different endstations.

To identify “hot pixels” on the LPD, as previously reported by Aleksich *et al.*,^57^ a series of masking procedures was performed. Briefly, before data collection, a crude mask was created based on peakfinder results from water and lysozyme crystals to eliminate peaks significantly above the average occurrence. Additionally, indexing results using the MOSFLM algorithm in CrystFEL^54^ on lysozyme were utilized. Pixels causing significant deviations in indexing at the extremes of standard lysozyme unit cell distributions were removed. This initial masking facilitated a reasonably accurate live hit rate determination through EXtra-Foam.^44^ Post-beamtime, for larger datasets, peakfinder and indexing results were revisited to remove pixels that showed peaks significantly above average. The mask is generated by an isolation forest algorithm. Specifically, we used the implementation from scikitlearn.^58^ The features that were fed to the algorithm are the differences between the mean, standard deviation, skewness, and kurtosis to its respective median value. Further detector masking was then informed by the peakogram output from CrystFEL.^54^ The peakogram distributions, represented as histograms of intensity and resolution over a run, were used to identify areas with non-continuous peak accumulation. The locations of these “hot areas” were then plotted on the detector to pinpoint additional masking zones, as detailed in the supplementary information Figs. E and F. Furthermore, the average intensity recorded for each pixel across all indexed Bragg peaks helped identify areas that were not correctly intensity-calibrated. We note that only 15 panels (out of 16) on the LPD detector were operational during our experiment. Ultimately, approximately 17% of the usable part of the LPD detector were masked.

## RESULTS

### Crystallographic results

**FIG. 3.**
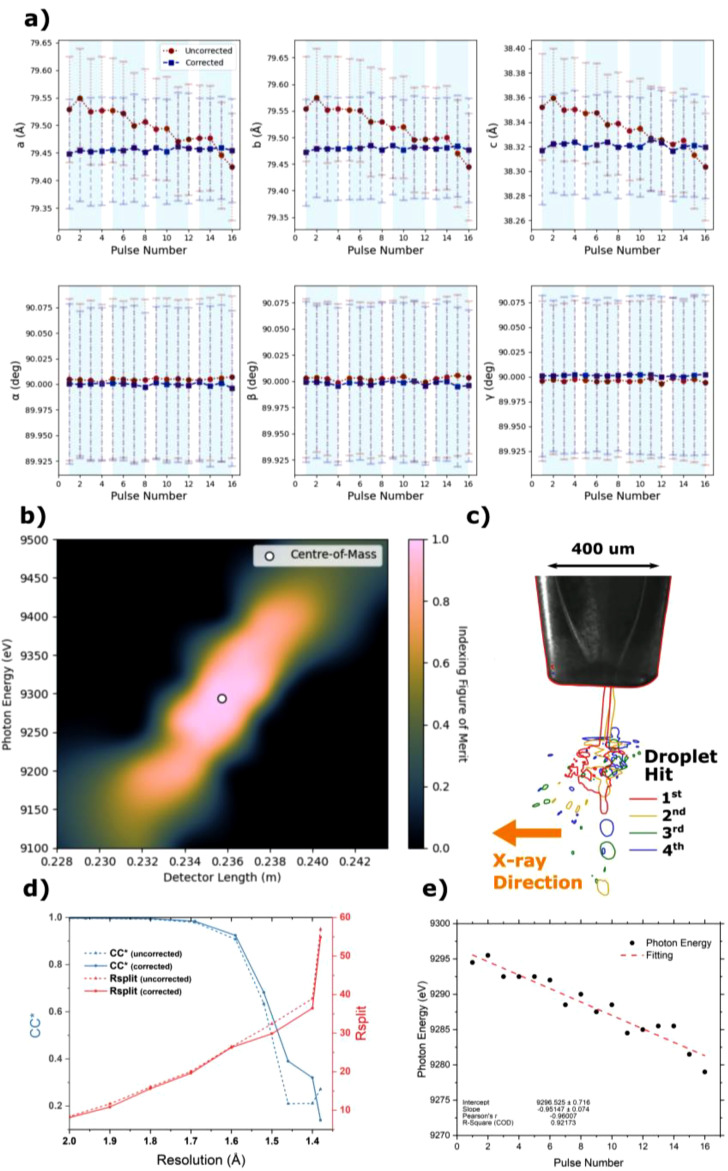
Photon energy drift and subsequent correction**.** (a) Unit cell dimensions for pulses over injection scheme, and points are centers of fitted Gaussians with errors being standard deviation of the fit supplementary information Fig. I. Red circles are values from indexing all pulses with the same photon energy (9290 eV), and blue squares are the results after fitting a photon energy to each pulse to maintain constant unit cell dimension (corrected data). Light blue background on plots indicate groups of droplets. (b) Indexing figure of merit for various photon energies and detector distances, see the main text for details. (c) Overlayed images of sequential hits traced in their respective color and show no obvious drift in the sample to detector distance. (d) Crystallographic merging statistics, CC^*^, and Rsplit, for various resolution shells show an improvement after photon energy correction. (**e)** Photon energy used to correct unit cell distribution for each pulse, a drift of ∼15.5 eV was observed over the train.

Initially, parameters, such as detector distance, beam center, and photon energy, were optimized to yield high indexing rates and symmetrical, low standard deviation unit cell distributions.^53^ Once processed, the data were separated according to pulse number within the train to check for variations in crystallographic statistics and structures. The unit cell distributions of each pulse were fitted with a Gaussian function using the relevant tool within CrystFEL's “cell_explorer.” Figure 3(a) displays the centers of these Gaussian fits in red along with their standard deviations (the raw histograms and fits can be found in supplementary information Fig. I). A noticeable trend of decreasing unit cell dimensions, of approximately 0.15% along axes a, b, and c, is observed over the course of the train. A changing unit cell dimension could be induced by a variety of factors, such as a varying sample-to-detector distance, droplet heating, or a drift in photon energy over the train. Given the large detector distance used in this experiment (236 mm), a change in the unit cell of 0.10 Å would necessitate a shift in the sample-to-detector distance of about 400 *μ*m over the duration of the train. It is unlikely that the interaction region systematically moves by 400 *μ*m in the direction of the XFEL beam, and also not supported by the jet imaging. Figure 3(c) traces the outlines of images of the explosion from every hit superimposed on top of each other. Since the camera was mounted perpendicular to the x-ray beam, any horizontal drift from hit-to-hit, and therefore, detector distance would be visible. However, no such trend is observed. Spatial or thermal trends from consecutive drops should reset with the introduction of each new Knackwurst and are therefore also unlikely to be the cause. Furthermore, heating would be expected to change the unit cell by enlargement rather than reduction, while we observe a decrease in unit cell dimensions.

These observations strongly suggest a systematic drift in the photon energy of the train. Unfortunately, accurate photon energy data were not recorded during the beamtime, so only a comparative photon energy reference can be used to correct one pulse relative to another. To address this, we employed a strategy where the first pulse within the train, which had the highest number of indexed frames, was used to optimize both the detector distance and photon energy. Subsequent pulses could then be corrected based on the parameters established during this optimization, assuming no change in the detector distance.

A figure of merit (FOM_indexing_) was defined to assess the quality of specific detector distance and photon energy combinations. This metric relied on two primary factors: the number of indexed patterns (IR) and the Gaussian quality of the unit cell distribution, with a focus on standard deviation (σ) and skewness (κ). In total, 638 different combinations of detector distance and photon energy were explored for indexing, resulting in a unique FOM for each combination. To characterize the quality of indexing, these FOM values were interpolated to create a 2D matrix and normalized, with the maximum value set to 1 [Fig. 3(b)]. The center-of-mass was calculated from this matrix to determine the ideal detector distance and photon energy, yielding unit cell dimensions of a = 79.45 Å, b = 79.47 Å, c = 38.32 Å, and α = β = γ = 90°. The non-interpolated individual indexing results are shown in supplementary information Fig. J. For each subsequent pulse, the photon energy was scanned to fit the unit cell dimensions to that of the first optimized pulse,

$${\mathrm{FOM}}_{\text{indexing}}=\mathrm{IR}\times \left(1-\sum_{i=a,b,c,\alpha \beta \gamma }\frac{\left|\sigma_{i}\kappa_{i}\right|\;}{6}\right).$$

The photon energy drift required to correct the unit cells over the train was 15.5 eV [Fig. 3(e)]. The corrected unit cell plot is shown in Fig. 3(a) with blue squares. The photon energy for each of the pulses is depicted in Fig. 3(e). It is noteworthy that the same amount of unit cell drift from the start to the end of the macropulse was observed at half the repetition rate. The drift remains within the Self-Amplified Spontaneous Emission (SASE) bandwidth of 25 eV.^59^ A similar magnitude of energy drift is observed in the study by Kujala *et al.* (Figs. 12 and 13).^59^ Although no explicit conclusion is provided, the energy drift appears to be correlated with the pulse intensity in the SASE process.^59^ Shot-to-shot x-ray photon energy via the HIgh REsolution hard X-ray single-shot spectrometer (HIREX) spectrometer would negate this issue and is recommended for SFX experiments conducted at EuXFEL.^59^ CrystFEL has the ability to apply a separate photon energy on a frame-by-frame basis. This correction is important for time-resolved SFX (TR-SFX) experiments where a very accurate estimate of the unit cell for the detection of small light induced differences. Figure 3(d) shows crystallographic merging statistics before and after the photon energy correction. A clear decrease in Rsplit and increase in CC^*^ are observed at higher resolutions, indicative of increase in crystallographic data quality.^60,61^

### Crystallographic comparison of sequential hits and droplets

Data from each pulse were indexed with the corrected photon energy as described above. All the data were then concatenated, scaled, and merged with CrystFEL's partialator, to ensure consistent scaling across all data. The stream files were merged either with point group 4/mmm for the standard case or 422 for the anomalous difference map (merging statistics for both are shown in Table I). In total, 74,048 indexed frames were collected over a 27 min period. R_free_ sets of 10% were then generated using CCP4i2's FreeRFlag.^63^ PHENIX's function phenix.refine^64^ was used for iterative rounds of model building based on a lysozyme model [Protein Data Bank (PDB)^65^ accession code 6H0K^47^]. The appropriate resolution cutoff for the crystallographic data was determined using Diederichs and Karplus “paired refinement.”^62,66,67^ This approach implements successive rounds of refinement including higher and higher resolution shells. A comparison of the R-factors is used to define when data at a certain resolution no longer contribute to a better model. The PAIREF package outputs a “strict” and “benevolent” cutoff, which, for the data presented here, was 1.5 and 1.38 Å, respectively.^62^ The full output from the refinement procedure is shown in supplementary information Figs. M and N. Notably, the Fourier shell correlation (FSC) is still significantly above 50% at the limiting resolution of 1.38 Å (supplementary information Fig. N).^69^ The resulting merging and refinement statistics for both cutoffs are given in Table I.

**FIG. 4.**
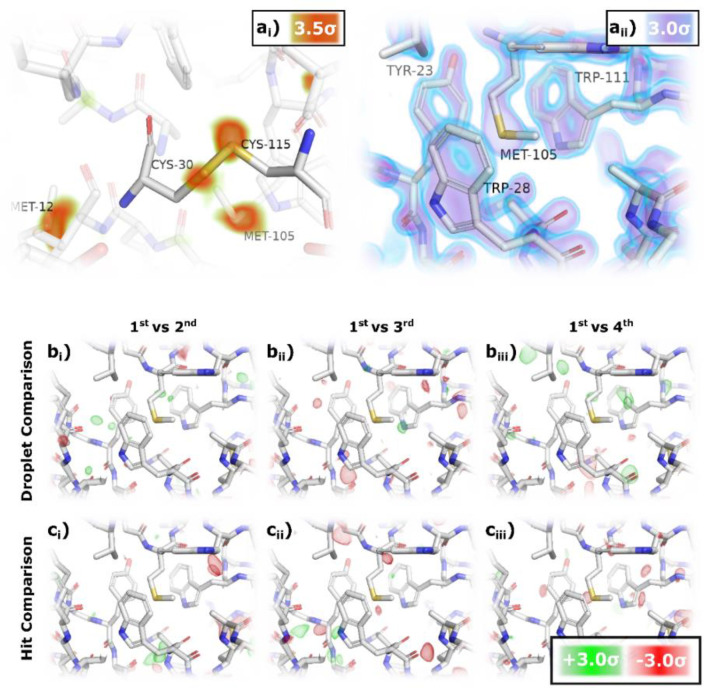
Electron density maps for Lysozyme. (a_i_) Phased anomalous Fourier difference map shown at 3.5 σ, highlighting sulfur atoms of various residues. (a_ii_) 2Fo-Fc map at 3σ (purple) to 3.5σ (blue) highlighting resolution of data with clear holes in density around aromatic groups (PDB: 8RUS). (b) and (c) Isomorphous difference maps at 3.0σ comparing all hits merged in the first Knackwurst to subsequent Knackwursts (b) and all Knackwursts merged for first hit to subsequent hits (c). No obvious difference density indicates no issues with hitting droplets multiple times kHz rates.

**TABLE I.** Lysozyme crystallographic and refinement statistics for merged runs 48,49, and 50 at EuXFEL beamtime p002808. Highest resolution shells for each strict and benevolent cutoff shown in parentheses at (a) 1.50–1.53 Å and (b) and (c) 1.38–1.40 Å. Resolution cutoff informed from paired refinement procedure PAIREF.^62^ Data merged in P4_3_2_1_2 (PDB 8RUS) for most of data processing except for the anomalous difference maps shown in Fig. 4(a_i_) merged P422, a non-centrosymmetric point group.

Data collection	
Wavelength (eV)	9295.5–9279
No. of indexed patterns	74 048

Merged statistics	P4_3_2_1_2		P422
	Strict (1.5 Å)	Benevolent (1.38 Å)	Anomalous maps
Resolution limit (A)	18.73–1.5	18.73–1.38	18.73–1.38
Total number of reflections	11 981 740 (322 575)	12 383 860 (107 809)	12 333 371
Number of unique reflections	20 240	25 840	48 251
Completeness	100 (100)^a^	100 (100)^b^	100 (99.98)^c^
Redundancy	591.0 (163.8)^a^	479.3 (42.7)^b^	255.6 (22.3)^c^
Signal to noise	8.34 (1.66)^a^	6.77 (0.90)^b^	5.07 (0.79)^c^
Wilson B-factor	30.43	25.21	25.09
Rsplit(%)	8.29 (29.30)^a^	8.36 (54.88)^b^	10.85 (75.67)^c^
CC^*^	0.997 (0.399)^a^	0.997 (0.134)^b^	0.996 (0.000)^c^
CC_1/2_	0.990 (0.086)^a^	0.990 (0.010)^b^	0.985 (0.001)^c^
	

Refinement statistics	P4_3_2_1_2		P422
	Strict (1.5 Å)	Benevolent (1.38 Å)	Anomalous maps
Resolution range	18.73–1.5	18.73–1.38	18.73–1.38
Point group	P 43 21 2	P 43 21 2	P 422
Unit cell	79.47, 79.47, 38.32, 90, 90, 90	79.47, 79.47, 38.32, 90, 90, 90	79.47, 79.47, 38.32, 90, 90, 90
Reflection for refinement	18 172	23 142	43 241
Reflections for R-free	2 023	2 639	4 899
No. none-hydrogen atoms	2 165	2 165	2 165
R-work	0.1595	0.1679	0.1489
R-free	0.1927	0.2015	0.1969
RMS (bonds)	0.009	0.009	0.008
RMS (angles)	1.036	1.002	0.932
Ramachandran favored (%)	99.21	99.21	99.21
Ramachandran allowed (%)	0.79	0.79	0.79
Ramachandran outliers (%)	0	0	0
Rotamer outliers (%)	0	0	0
Clash-score	3.8	2.9	1.44
Average B-factors	38.6	35.6	38.0

The high-quality crystallographic data result in the electron density maps shown in Fig. 4 and are displayed in PyMOL.^68^ Figure 4(a_i_) displays an anomalous Fourier difference map at 9.3 keV, revealing clear density on the sulfur atoms contoured at 3.5σ. Furthermore, the density is localized on the individual atoms within the disulfide bond between CYS-30 and CYS-115. Clear density on the sulfurs indicates good signal-to-noise ratio, as this experiment was conducted with 9.3 keV, far from sulfur's K-edge absorption at 2.4 keV, and hence, sulfur has a minimal cross section at this wavelength. In Fig. 4(a_ii_), the 2Fo-Fc map of the data, cutoff to 1.38 Å, displays clear density for the aromatic groups (PDB: 8RUS). Figures 4(b) and 4(c) report isomorphous difference maps at ±3σ, comparing the first Knackwurst to subsequent Knackwurst and the first hit to within a Knackwurst to the subsequent hits. No interpretable features were found in any difference map, with the limited visible density attributed to noise.

### Data rate and sample consumption

The high hit rate of the DoD method means that, despite not running at the MHz repetition rates of the GDVN injection methods, the actual data rate, which we define as the indexed frames per second, is still competitive. Table II is a comparison of published datasets taken at EuXFEL on lysozyme. The data rate achieved in this work is second to only one megahertz study.^37^ We note, however, that the work presented here was a time-limited testing at the start of a beamtime, and it is not unreasonable to imagine further improvement of this rate with more time and optimization.

**TABLE II.** Comparison of published lysozyme data collected at EuXFEL. Highlighting the data rate, quality, and low sample consumption of a DoD. All parameters calculated from most conservative values listed in various publications.

Delivery method	Intra train repetition rate (MHz)	Effective repetition rate (kHz)	Data rate(Indexed frames per second)	Resolution (Å)	Sample consumption(mg/min)	Sample over beamtime^a^

GDVN^37^	1.2	1.2	99	1.6	1.8	0.303
GDVN^38^	1.125	0.15	5.1	1.7	1.8	5.882
GDVN^47^	1.125	0.5	11	1.9	1.75	2.727
GDVN^39^	1.125	0.14	3.1	2.1	1.64	8.817
GDVN^40^	1.1	0.3	10.4	2	⋯	⋯
DoD	0.047	0.16	45.7	1.5 (1.38)	0.0175	0.006

^a^ Assuming 1 000,000 indexed frames and no sample wastage (grams).

The DoD method minimizes the sample volume required for an SFX experiment, particularly for the EuXFEL macro-pulse structure because there is no sample waste. Table II presents the sample consumption rates of various lysozyme SFX experiments at EuXFEL, which shows that the DoD's consumption rate is two orders of magnitude lower than GDVN. Once optimized, the DoD method can reliably deliver samples over an extended period without clogging. Furthermore, sample changeovers are less frequent due to the lower consumption rate. In this work, a single load would last approximately 1-2 h, compared to the more common 30 min for GDVN. This duration could be extended further, limited only by crystal settling in the sample delivery tube. Table II additionally lists the amount of sample required for 1 × 10^6^ indexed frames, which is typical of a very successful TR-SFX experiment. This reduces the amount of protein required for an SFX experiment from the grams to milligrams scale, making this structural measurement much more feasible for a vast range of proteins.

We obtain for the first-time high-quality protein crystallographic data from the LPD that was not designed for crystallography. The high-resolution data are facilitated by the large detector area of the LPD and the short distance between the detector and the sample, which is adaptable with the movable LPD—unlike other endstations with fixed distances. The quality of the data benefits from the LPD's extensive dynamic range (1:100 000 photons), which tolerates a higher x-ray flux, and the partial helium environment that lowers the signal-to-noise ratio. We expect data quality was degraded with the scatter from the larger liquid volume of the droplets of the DoD. Further reductions in sample consumption and noise could be achieved by using a smaller orifice size. To improve future experiments, it would be beneficial to reduce the amount of air between the sample and detector, for example, using a helium bag.

## CONCLUSIONS

This work demonstrates kHz droplet-on-demand injection at the EuXFEL, achieving data rates competitive with current state-of-the-art MHz GDVN sample delivery methods. This approach significantly reduces the volume of consumed sample by two orders of magnitude over GDVN.

A systematic drift in the average photon energy over the train was observed, and correcting this chirp improved the crystallographic data. We develop methods to process SFX data from the LPD detector that result in excellent crystallographic statistics and high-quality structure. We report the highest resolution protein structure collected at EuXFEL (1.38 Å), attributed to using a smaller detector distance with a large dynamic range of the LPD, used for the first protein crystallography experiment at the FXE endstation.

It is anticipated that SFX experiments performed at this facility will benefit from shot-to-shot photon energy diagnostics. Comparison of multiple droplet hits showed no significant changes over the train, making the DoD a suitable injection method at these rates. With advancements in inkjet technology injectors, it is predicted that the limitations of current DoD setups will decrease, approaching megahertz droplet injection rates.^35^ The reduced sample consumption, while maintaining high crystallographic quality data, will make SFX beamtimes more feasible for a wider range of biologically and chemically relevant samples.

## Data Availability

The data that support the findings of this study are available from the corresponding author upon reasonable request.
